# The High Level of Xylooligosaccharides Improves Growth Performance in Weaned Piglets by Increasing Antioxidant Activity, Enhancing Immune Function, and Modulating Gut Microbiota

**DOI:** 10.3389/fnut.2021.764556

**Published:** 2021-12-06

**Authors:** Jiaman Pang, Xingjian Zhou, Hao Ye, Yujun Wu, Zhenyu Wang, Dongdong Lu, Junjun Wang, Dandan Han

**Affiliations:** ^1^State Key Laboratory of Animal Nutrition, College of Animal Science and Technology, China Agricultural University, Beijing, China; ^2^Adaptation Physiology Group, Department of Animal Sciences, Wageningen University and Research, Wageningen, Netherlands

**Keywords:** Xylooligosaccharides, weaning piglets, growth performance, antioxidant, immune function, fecal microbiota, short-chain fatty acids

## Abstract

The aim of this study was to investigate the effects of the high level of xylooligosaccharides (XOS) on growth performance, antioxidant capability, immune function, and fecal microbiota in weaning piglets. The results showed that 28 d body weight exhibited linear and quadratic increases (*P* < 0.05) with increasing dietary XOS level, as well as average daily feed intake (ADFI) on d 15–28, average daily gain (ADG) on d 15–28 and 1–28. There was a linear decrease (*P* < 0.05) between XOS levels and feed conversion rate (FCR) on d 1–14 and 1–28. Additionally, glutathione peroxidase (GSH-Px) showed a linear increase (*P* < 0.05), while the malondialdehyde (MDA) level decreased linearly and quadratically (*P* < 0.05) with the increasing dietary level of XOS. Moreover, the XOS treatments markedly increased the levels of immunoglobulin A (Ig A) (linear, *P* < 0.05; quadratic, *P* < 0.05), IgM (quadratic, *P* < 0.05), IgG (linear, *P* < 0.05), and anti-inflammatory cytokine interleukin-10 (IL-10) (quadratic, *P* < 0.05) in serum, while the IL-1β (linear, *P* < 0.05; quadratic, *P* < 0.05) and IL-6 (linear, *P* < 0.05) decreased with increasing level of XOS. Microbiota analysis showed that dietary supplementation with 1.5% XOS decreased (*P* < 0.05) the α-diversity and enriched (*P* < 0.05) beneficial bacteria including *Lactobacillu*s, *Bifidobacterium*, and *Fusicatenibacter* at the genus level, compared with the control group. Importantly, linearly increasing responses (*P* < 0.05) to fecal acetate, propionate, butyrate, and total short-chain fatty acids (SCFAs) were observed with increasing level of XOS. Spearman correlation analyses found that *Lactobacillu*s abundance was positively correlated with ADG, acetate, propionate, and IgA (*P* < 0.05), but negatively correlated with IL-1β (*P* < 0.05). *Bifidobacterium* abundance was positively related with ADFI, total SCFAs, IgG, and IL-10 (*P* < 0.05), as well as *g*_*Fusicatenibacter* abundance with ADFI, total SCFAs, and IL-10. However, *Bifidobacterium* and *Fusicatenibacter* abundances were negatively associated with MDA levels (*P* < 0.05). In summary, dietary supplementation with XOS can improve the growth performance in weaning piglets by increasing antioxidant capability, enhancing immune function, and promoting beneficial bacteria counts.

## Introduction

In modern swine production, weaning is a critical period for piglets to encounter multiple stressors including environmental, dietary, and social changes (1). Due to immunological and physiological immaturity, weaning results in stress syndromes in piglets including transient anorexia, unbalanced gut microbiota, increased diarrhea incidence, and decreased growth performance (2). China is the biggest porker producer and consumer in the world but about 24 million piglets die from diarrhea caused by bacterial infection after weaning each year (3, 4). Thus, antibiotics were widely applied to prevent pathogenic infection and promote growth in the postweaning period (5). However, the antibiotic growth promoters have been limited to being used in swine industry in China due to the adverse effects of antibiotics resistance and residue, which pose a great threat to human health and food security (3). To maintain piglet health during the weaning period and preserve public health, it is urgent to find and develop antibiotic alternatives.

The growing evidence suggested prebiotics as preferable alternatives to antibiotics (6). Prebiotics are non-digestible substrates that improve host health by selectively stimulating growth or activity of specific bacteria (7). At present, commercial prebiotics are mainly functional oligosaccharides, such as galactooligosaccharides (GOS), mannanoligosaccharides (MOS), fructooligosaccharides (FOS), and xylooligosaccharides (XOS) (5, 6). The XOS are produced from hydrolysis of xylan, which widely exist in plant cell wall and various agricultural waste or byproducts. The XOS are composed of 2–10 xylose monomers linked through β-(1,4) linkages with acidity and temperature stability, and non-toxicity (8–10). Many researches indicated that dietary supplementation of XOS improve the animal growth performance in weaning piglets and broiler chicken by enhancing immune functions, elevating antioxidant activity, increasing nutrient digestibility, and maintaining intestinal morphology and barrier (4, 11–14). In addition, some studies found that XOS are able to increase short-chain fatty acids (SCFAs) concentrations in broiler chicken or weaning piglets (4, 15), stimulate proliferation of *Lactobacillu*s, and inhibit the growth of *Escherichia coli* in weaning piglets (4, 16). Christensen et al. (17) found that *Bifidobacteriaceae* varied approximately 10,000-fold from 0.001 to 10.7% in rats after consumption of 10% XOS. However, the abundance of *Bifidobacterium* had no remarkable increase at the low dosage of XOS in pigs (4, 11, 18).

Whether the high level of XOS can promote *Bifidobacterium* microbiota in weaning piglets remains unknown. Meanwhile, the effects of the high level of XOS on growth performance, antioxidant capability, and immune function have not been fully studied in weaning piglets. Therefore, the objective of this study was to investigate the high level of XOS effects on growth performance, antioxidant capability, and anti-inflammatory action, and whether the high level of XOS promote *Bifidobacterium* abundance of weaned piglets.

## Materials and Methods

This work was approved by the Animal Ethics Committee of China Agricultural University.

### Animals, Housing, and Experimental Design

A total of 192 piglets (Duroc × Landrace × Large White) weaned at 30 d of age with an average initial body weight (BW) of 7.50 ± 0.8 kg were randomly divided into four groups based on BW and sex. Each group had six replicate pens with eight piglets per pen. The control group piglets were fed with a control diet without the supplementation of XOS. The XOS-treated groups piglets were received dietary supplementation of 0.75% XOS, 1.5% XOS, and 3% XOS, respectively. The XOS (P70, 70%) were provided by Longlive Biotechnology Corporation (Shandong, China). The experimental period included two feeding phases: phase 1, from d 1 to d 14 postweaning; phase 2, from d 15 to d 28 postweaning. The dietary compositions and nutrient concentrations are presented in Table 1. All diets without antibiotics were formulated to meet the nutrient requirements of National Research Council (19). The pigs were housed in an environmentally controlled room with slatted plastic flooring. The environmental temperature was maintained at 26–28°C and relative humidity was controlled at 40–50%. The pigs were given *ad libitum* access to feed and water throughout the trial for 28 days.

**Table 1:** Composition of the experimental diets (as-fed basis).

**Items**	**Phase 1 (1–14 d), XOS (% of diet)**				**Phase 2 (15–28 d), XOS (% of diet)**			
	**Control (0)**	**0.75**	**1.5**	**3**	**Control (0)**	**0.75**	**1.5**	**3**
**Ingredient (%)**								
Corn	61.95	60.65	59.15	56.70	67.16	65.76	64.41	62.06
Soybean meal 43%	10.00	10.00	10.00	10.00	15.00	15.00	15.00	15.00
Dehulled soybean meal 46%	5.00	5.20	5.50	5.90	2.00	2.30	2.50	2.90
Fish meal	5.00	5.00	5.00	5.00	3.00	3.00	3.00	3.00
Expanded soybean	6.00	6.00	6.00	6.00	6.00	6.00	6.00	6.00
Whey power	8.00	8.00	8.00	8.00	3.00	3.00	3.00	3.00
Soybean oil	0.40	0.75	1.20	1.75	0.40	0.75	1.15	1.60
XOS	0.00	0.75	1.50	3.00	0.00	0.75	1.50	3.00
CaHPO_4_	1.15	1.15	1.15	1.15	1.10	1.10	1.10	1.10
Limestone	0.80	0.80	0.80	0.80	0.70	0.70	0.70	0.70
Salt	0.20	0.20	0.20	0.20	0.20	0.20	0.20	0.20
Primix^a^	0.50	0.50	0.50	0.50	0.50	0.50	0.50	0.50
*L*-Lys-HCl (78.8%)	0.61	0.61	0.61	0.61	0.55	0.55	0.55	0.55
*DL*-Met (98%)	0.12	0.12	0.12	0.12	0.12	0.12	0.12	0.12
*L*-Thr (98%)	0.22	0.22	0.22	0.22	0.22	0.22	0.22	0.22
*L*-Trp (98%)	0.05	0.05	0.05	0.05	0.05	0.05	0.05	0.05
Total	100.00	100.00	100.00	100.00	100.00	100.00	100.00	100.00
**Calculated nutrient level (%)**								
Digestible energy (MJ/kg)	14.44	14.41	14.41	14.42	14.45	14.42	14.40	14.42
Crude protein (%)	18.98	18.97	18.99	18.98	18.28	18.31	18.29	18.29
Total Ca (%)	0.91	0.91	0.91	0.91	0.75	0.75	0.75	0.75
Total *P* (%)	0.71	0.71	0.71	0.71	0.63	0.63	0.63	0.63
Lys (%)	1.35	1.35	1.35	1.36	1.24	1.25	1.25	1.26
Met (%)	0.40	0.40	0.40	0.40	0.38	0.38	0.38	0.38
Thr (%)	0.80	0.80	0.80	0.80	0.76	0.76	0.76	0.76
Trp (%)	0.23	0.23	0.23	0.23	0.22	0.22	0.22	0.22

a *The premix provided the following per kg of diets: VA 12,000 IU, VD_3_ 2,500 IU, VE 30 IU, VK_3_ 3 mg, VB_5_ 10 mg, VB_12_ 27.6 μg, niacin 30 mg, choline chloride 400 mg, Mn (as MnO) 40 mg, Fe (as FeSO_4_·H_2_O) 90 mg, Zn (as ZnO) 100 mg, Cu (as CuSO_4_·5H_2_O) 8.8 mg, I (as KI) 0.35 mg, Se (Na_2_SeO_3_) 0.3 mg*.

### Samples Collection

Individual pig BW was recorded at the initial of the weaning and on d 14 and d 28. Pen feed intake was also measured on d 14 and d 28 of this experiment. The average daily gain (ADG), average daily feed intake (ADFI), and feed conversion rate (FCR) were calculated. The feces of pigs were scored daily for diarrhea according to the following criteria: 1 = firm and well-form feces, 2 = soft and form feces, 3 = sloppy feces and mild diarrhea, and 4 = pasty and liquid diarrhea. The incidence of diarrhea was calculated for the first 7 d after weaning. The incidence of diarrhea was calculated as follows: Diarrhea incidence (%) = (total number of pigs per pen with diarrhea)/(number of pigs per pen × 7 d) × 100%.

On d 28, eight pigs of each group were randomly selected (at least one pig per pen) to collect blood samples from precaval vein into 10 mL sterile tubes. The blood samples were centrifuged at 3,000 × g at room temperature for 10 min to obtain serum, and stored at −80°C for further analysis. Fecal samples (one pig per pen) were collected into sterile tubes on d 28 and stored at −80°C for further analysis.

### Biochemical and Immunological Parameters in Blood

Serum total protein (TP), albumin (ALB), albumin globulin ratio (A/G), alanine aminotransferase (ALT), aspartate aminotransferase (AST), blood urea nitrogen (BUN), and alkaline phosphatase (ALP) were measured using automatic biochemical analyzer (Hitachi 7020, Tokyo, Japan). The total superoxide dismutase (T-SOD) activity, catalase (CAT) activity, total antioxidant capacity (T-AOC), malondialdehyde (MDA) content, and glutathione peroxidase (GSH-Px) capacity in serum were measured using commercial kits (Jiancheng Co., Ltd, Nanjing, China). The activity of T-SOD was determined at 450 nm by the xanthine oxidase method. The CAT activity was determined at 405 nm by ammonium molybdate method. The T-AOC was determined at 405 nm by 2,2′-azinobis-(3-ethylbenzthiazoline-6-sulphonate) method. The MDA content was measured at 532 nm using the thiobarbituric acid method. The activity of GSH-Px was measured at 412 nm using 2,2′-dithiodibenzoic acid method. The levels of immunoglobulin A (IgA), IgG, IgM, interleukin-1β (IL-1β), IL-6, and IL-10 in serum were detected by commercial ELISA kits (Jiancheng Co., Ltd, Nanjing, China) according to manufacturer's instructions.

### Analysis of Microbiota in Feces

The total bacterial DNA of the feces was extracted using the QIAamp Fast DNA Stool Mini Kit (Qiagen, Germany) following the manufacturer's protocol. The V3–V4 hypervariable regions of bacteria 16S rDNA were amplified with universal primers 338F (ACTCCTACGGGAGGCAGCAG) and 806R (GGACTACHVGGGTWTCTAAT). The purified PCR products were equimolarly combined and paired-end sequenced on the Illumina Miseq platform (Illumina, San Diego, CA, USA).

The raw reads were demultiplexed and quality filtered by Quantitative Insights Into Microbial Ecology (QIIME) (version 1.9) software with the following criteria: (i) the reads were truncated at any site receiving an average quality score of <20 over a 50 bp sliding window, and the truncated reads shorter than 50 bp were discarded, reads containing ambiguous characters were also discarded; (ii) only overlapping sequences longer than 10 bp and the reads without more than two nucleotide mismatches in the primer were assembled. The remaining sequences with 97% similarity were clustered into the same operational taxonomic units (OTUs) using UPARSE (version 7.1) and chimeric sequences were removed. The taxonomy of each OTU representative sequence was analyzed by Ribosomal Database Project (RDP) classifier against the SILVA 16S rRNA gene database using confidence threshold of 70%. The analysis of the α-diversity was calculated by the MOTHUR program. For the β-diversity, principal coordinate analysis (PCoA) was performed based on Bray-Curtis distances. The data were analyzed on the Majorbio I-Sanger cloud platform (www.i-sanger.com).

### Quantification of Specific Bacteria in Feces

The total bacterial DNA of feces was isolated as described above. The absolute copy numbers of specific bacteria were calculated as previously described (22). The primers were listed in Table 2.

**Table 2:** Specific bacteria primers used in this study.

**Primer**	**Forward 5′ → 3′**	**Reverse 5′ → 3′**	**Reference**
Total bacteria	ACTCCTACGGGAGGCAGCAG	ATTACCGCGGCTGCTGG	(20)
*Bifidobacterium* spp.	CGCGTCCGGTGTGAAAG	CTTCCCGATATCTACACATTCCA	
*Lactobacillus* spp.	GAGGCAGCAGTAGGGAATCTTC	GGCCAGTTACTACCTCTATCCTTCTTC	
*E. coli*	CATGCCGCGTGTATGAAGAA	CGGGTAACGTCAATGAGCAAA	
*Salmonella* spp.	CCTTTCTCCATCGTCCTGAA	TGGTGTTATCTGCCCGACCA	(21)

### Determination of SCFAs in Feces

The concentrations of SCFAs, including lactate, acetate, propionate, and butyrate in the feces were determined using Ion Chromatograph. Briefly, 0.5 g of feces was weighed and dissolved in 10 mL distilled water, homogenized using ultrasound for 30 min at room temperature, and centrifuged at 5,000 × g for 10 min to obtain the supernatants. The supernatants were diluted 10 × and filtered using 0.22 μm membranes before being detected by high-performance ion chromatography system (DIONEX ICS-3000; Thermo Fisher, USA). The injection volume was 25 μL and the flow rate was 1.0 mL/min (23).

### Statistical Analysis

All the data were performed using Statistics Analysis System (SAS) 9.4 version (Cary, NC, United States). Data statistical significances were analyzed by one-way ANOVA for multiple groups. Statistical differences among the treatments were separated by Duncan's multiple tests method. The data of two groups were analyzed by unpaired Student's *t*-test. The incidence of diarrhea was analyzed by χ^2^ test. The differential bacteria were identified by using linear discriminant analysis (LDA) effect size (LEfSe) analysis. The level of statistical significance was *P* ≤ 0.05, whereas 0.05 < *P* < 0.1 was considered as a trend of significant difference. Data are presented as means ± SEM.

## Results

### Effects of XOS Supplementation on Growth Performance in Weaning Piglets

The dietary supplementation with the high level of XOS had no effects (*P* > 0.05) on 14 d BW, ADG on d 1–14, ADFI on d 1–14, and FCR on d 15–28 and 1–28 (Table 3). However, supplementation with XOS significantly increased ADG on d 15–28 and 1–28 (*P* < 0.05). Similarly, 28 d BW in 1.5% and 3% XOS groups had higher than the control group (*P* < 0.05), as well as ADFI on d 15–28. Notably, 1.5% XOS group markedly increased (*P* < 0.05) ADFI on d 1–28 compared with other groups, but there were no differences among the other groups (*P* > 0.05). Compared with the control group, the FCR on d 1–14 was markedly decreased in the 3% XOS group (*P* < 0.05). In addition, linear and quadratic increases (*P* < 0.05) were observed between different XOS levels and 28 d BW, as well as ADG on d 15–28 and 1–28, and ADFI on d 15–28 (Table 3), with the highest (*P* < 0.05) at the supplemental level of 1.5% XOS. ADFI on d 1–14 and 1–28 exhibited quadratic increases (*P* < 0.05) with the increasing dietary level of XOS. There was a linear decrease (*P* < 0.05) between the dietary XOS levels and FCR on d 1–14 and 1–28, with the lowest FCR (*P* < 0.05) on d 1–14 at the level of 3% XOS (Table 3).

**Table 3:** The effects of XOS supplementation on growth performance of weaning piglets.

**Items**	**XOS (% of diet)**					***P*** **-valve**		
	**Control (0)**	**0.75**	**1.5**	**3**	**SEM**	**Diet**	**Liner**	**Quadratic**
Initial BW (kg)	7.50	7.52	7.53	7.50	0.10	0.999		
14 d BW (kg)	10.77	10.85	11.32	11.08	0.12	0.364	0.272	0.292
28 d BW (kg)	16.30^c^	16.84^bc^	17.70^a^	17.17^ab^	0.14	0.001	0.007	0.003
d 1–14								
ADG (g/d)	233.99	238.18	270.34	256.29	5.86	0.093	0.093	0.157
ADFI (g/d)	341.01	355.23	378.74	338.97	6.75	0.131	0.878	0.029
FCR	1.46^a^	1.50^a^	1.40^ab^	1.33^b^	0.02	0.037	0.011	0.511
d 15–28								
ADG (g/d)	394.79^c^	427.91^b^	455.75^a^	434.82^ab^	5.68	<0.001	0.001	<0.001
ADFI (g/d)	690.92^c^	717.22^bc^	797.56^a^	738.90^b^	10.15	<0.001	0.007	<0.001
FCR	1.75	1.68	1.75	1.70	0.02	0.400	0.510	0.932
d 1–28								
ADG (g/d)	314.39^c^	333.05^b^	363.05^a^	345.56^b^	4.53	<0.001	<0.001	<0.001
ADFI (g/d)	515.97^b^	536.23^b^	588.15^a^	538.94^b^	7.44	0.001	0.099	0.001
FCR	1.64	1.61	1.62	1.56	0.01	0.147	0.032	0.838
Diarrhea incidence (%)	12.50^a^	6.25^b^	7.74^b^	8.33^ab*^		0.035		

*BW, body weight; ADG, average daily gain; ADFI, average daily feed intake; FCR, feed conversion rate*.

Values with different letter superscripts mean significant difference (P < 0.05) in a row, while with no letter superscripts mean no significant difference (P > 0.05). *indicates a trend of significance (0.05 ≤ P < 0.1). The same as below. N = 6 for each group.

### Effects of XOS Supplementation on Diarrhea Incidence of Weaning Piglets

There was a decreasing trend for the incidence of diarrhea between the pigs fed with the control diet and the 3% XOS diet. However, dietary supplementation with 0.75% XOS and 1.5% XOS significantly decreased (*P* < 0.05) the diarrhea incidence compared with the control group in weaning piglets (Table 3).

### Effects of XOS Supplementation on Serum Biochemical Parameters and Antioxidant Indictors in Weaning Piglets

The serum biochemical parameters on d 28 are presented in Table 4. The XOS treatments had no effects on serum biochemical parameters on d 28 in weaning pigs (Table 4).

**Table 4:** The effects of XOS supplementation on serum biochemical parameters of weaning piglets.

**Items**	**XOS (% of diet)**					***P*** **-valve**		
	**Control (0)**	**0.75**	**1.5**	**3**	**SEM**	**Diet**	**Liner**	**Quadratic**
TP (g/L)	47.36	49.20	49.45	48.59	0.60	0.632	0.603	0.247
ALB (g/L)	35.94	36.31	35.71	35.81	0.51	0.981	0.847	0.981
GLB (g/L)	11.43	12.89	12.28	12.78	0.52	0.743	0.487	0.666
A/G	3.46	3.00	3.12	2.85	0.18	0.694	0.320	0.746
ALT (U/L)	40.06	44.95	39.39	39.44	1.15	0.258	0.437	0.541
AST (U/L)	43.25	41.85	44.53	39.13	2.15	0.851	0.545	0.657
ALP (U/L)	415.39	383.53	392.40	378.14	15.63	0.853	0.495	0.757
BUN (mmol/L)	2.73	2.83	2.59	2.51	0.13	0.829	0.434	0.626

*TP, total protein; ALB, albumin; GLB, globulin; A/G, albumin to globulin ratio; ALT, alanine aminotransferase; AST, aspartate aminotransferase; ALP, alkaline phosphatase; BUN, blood urea nitrogen; n = 6–8 for each group*.

The serum T-SOD, CAT, T-AOC, GSH-PX, and MDA were measured to evaluate the effects of XOS on antioxidant capability (Table 5). The results showed that dietary supplementation with XOS failed to affect T-SOD, CAT, and T-AOC activities (*P* > 0.05) except for GSH-Px and MDA (*P* < 0.05). Compared with the control group, both 1.5 and 3% XOS groups significantly increased the GSH-Px level. However, the XOS treatments significantly declined (*P* < 0.05) the MDA level compared with the control group, and the diet supplemented with 1.5% XOS (*P* < 0.05) had the lowest MDA concentration among the four groups. Besides, the GSH-Px showed a linear increase (*P* < 0.05) with the increasing dietary level of XOS, but MDA levels decreased linearly and quadratically (*P* < 0.05).

**Table 5:** The effects of XOS supplementation on antioxidant activities of weaning piglets.

**Items**	**XOS (% of diet)**					***P*** **-valve**		
	**Control (0)**	**0.75**	**1.5**	**3**	**SEM**	**Diet**	**Liner**	**Quadratic**
T-SOD (U/mL)	135.11	135.11	140.03	138.62	1.61	0.634	0.357	0.597
CAT (U/mL)	2.20	2.18	2.76	2.44	0.15	0.479	0.426	0.387
T-AOC (mmol/L)	0.23	0.24	0.25	0.24	0.004	0.317	0.406	0.162
GSH-Px (U/mL)	284.06^b^	293.75^ab^	312.19^a^	317.58^a^	4.65	0.030	0.005	0.355
MDA (nmol/mL)	3.86^a^	3.37^b^	2.70^c^	3.25^b^	0.11	0.001	0.017	0.001

*SOD, superoxide dismutase; CAT, catalase; GSH-Px, glutathione peroxidase; T-AOC, total antioxidant capacity; MDA, malondialdehyde. N = 6–8 for each group*.

*Values with different letter superscripts mean significant difference (P <0.05) in a row, while with no letter superscripts mean no significant difference (P > 0.05)*.

### Effects of XOS Supplementation on Immunological Function and Inflammatory Cytokines in Weaning Piglets

The serum levels of IgM, IgG, IgA, IL-1β, IL-6, and IL-10 were determined to evaluate the effects of XOS on immune status. As shown in Table 6, quadratic responses (*P* < 0.05) in serum levels of IgM and IL-10 were observed with the increasing supplemental XOS level. Addition of 0.75% XOS and 1.5% XOS had higher (*P* < 0.05) level of IgM than the control group. 1.5% XOS group also significantly increased IL-10 level compared with control group (*P* < 0.05). There were linear responses in serum levels of IgG and IL-6 (*P* < 0.05) with the increasing supplemental XOS level in the diets. Compared with the control group, 1.5 and 3% XOS groups significantly increased (*P* < 0.05) the IgG concentration in serum. However, the XOS-treated pigs remarkedly decreased (*P* < 0.05) serum level of IL-6, while no differences (*P* > 0.05) were found in XOS treatments. Additionally, IL-1β showed linear and quadratic (*P* < 0.05) responses with the increasing level of XOS in the diets, with the lowest (*P* < 0.05) serum concentration of IL-1β at the supplemental level of 1.5% XOS. Moreover, the XOS treatments significantly increased (*P* < 0.05) the level of IgA compared with control groups. The different levels of XOS showed linear and quadratic (*P* < 0.05) effects on IgA.

**Table 6:** The effects of XOS supplementation on immunological function and inflammatory cytokines of weaning piglets.

**Items**	**XOS (% of diet)**					***P*** **-valve**		
	**Control (0)**	**0.75**	**1.5**	**3**	**SEM**	**Diet**	**Liner**	**Quadratic**
IgM (g/L)	0.82^b^	0.92^a^	0.94^a^	0.87^ab^	0.02	0.011	0.488	0.001
IgG (g/L)	6.18^b^	6.48^ab^	7.05^a^	7.21^a^	0.14	0.029	0.006	0.350
IgA (g/L)	0.85^c^	1.00^b^	1.15^a^	1.15^a^	0.03	<0.001	<0.001	0.016
IL-1β (pg/mL)	21.74^a^	20.20^ab^	16.20^c^	18.89^b^	0.52	<0.001	0.008	0.001
IL-6 (pg/mL)	97.01^a^	80.21^b^	78.53^b^	84.13^b^	2.03	0.001	0.005	0.101
IL-10 (pg/mL)	22.91^b^	26.02^b^	30.50^a^	23.51^b^	0.75	<0.001	0.779	<0.001

*IgM, immunoglobulin M, IgG, immunoglobulin G, IgA, immunoglobulin A, IL-1β, interleukin-1β, IL-6, interleukin-6, IL-10, interleukin-10. N = 7–8 for each group*.

*Values with different letter superscripts mean significant difference (P < 0.05) in a row, while with no letter superscripts mean no significant difference (P > 0.05)*.

### Effects of XOS Supplementation on Fecal Microbiota in Weaning Piglets

Next, the microbiota of fecal samples in the control group and 1.5% XOS group was analyzed. As shown in Table 7, the alpha diversity was evaluated. The Shannon index had a significant decrease (*P* < 0.05) in the 1.5% XOS group compared with the control group, whereas the Simpson index had a significant increase in the 1.5% XOS group (*P* < 0.05). However, there were no impacts of 1.5% XOS on Sobs (*P* > 0.05), Ace, and Chao indices compared with the control group.

**Table 7:** The effects of XOS supplementation on fecal microbial diversity of weaning piglets.

**Items**	**Control**	**1.5% XOS**	**SEM**	***P*-value**
Sobs	330.00	313.67	9.05	0.392
Shannon	4.18	3.59	0.13	0.011
Simpson	0.04	0.09	0.01	0.026
Ace	347.35	333.95	7.30	0.384
Chao	356.77	340.55	7.85	0.324

*Fecal microbial α-diversity indices were analyzed by unpaired Student's t-test (n = 6)*.

**Figure 1:**
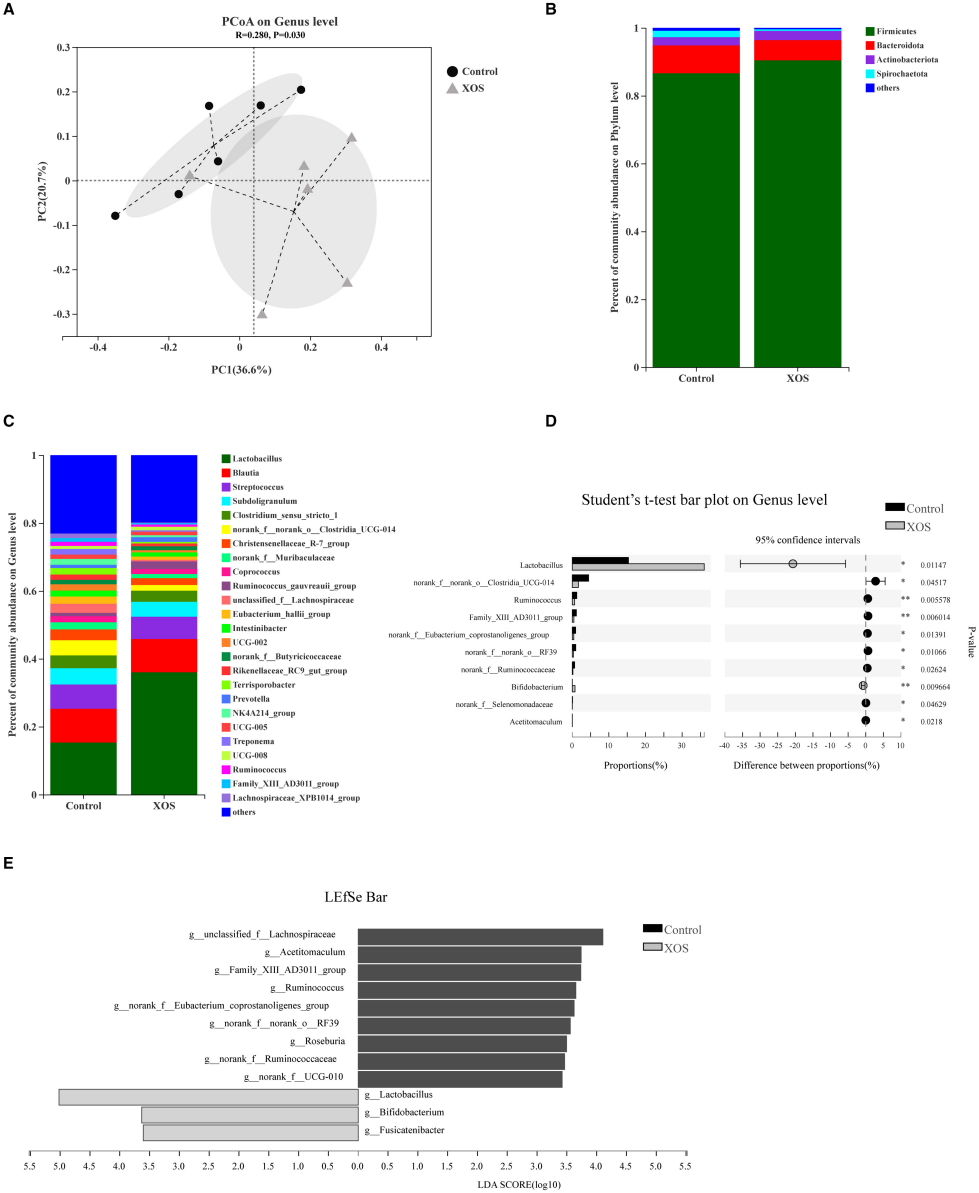
Dietary supplementation with xylooligosaccharides (XOS) modulates the composition of fecal microbiota. **(A)** Principal coordinate analysis (PCoA) on genus level based on Bray-Curtis distances. Composition of bacterial communities **(B)** at the phylum level and **(C)** at the genus level. **(D)** Differential bacteria at the genus level. **(E)** Linear discriminant analysis (LDA) effect size (LEfSe) analysis screened biomarker of microbial community after addition of XOS. *P < 0.05 and **P < 0.01, *n* = 6 for each group.

Bata-diversity was assessed using PCoA plots based on Bray-Curtis distances. The fecal microbiota composition in the 1.5% XOS group could obviously separate from the control group (*P* < 0.05, Figure 1A). At the phylum level, *Firmicutes, Bacteroidota*, and *Actinobacteriota* predominated in feces (Figure 1B). At the genus level, *Lactobacillu*s was dominant in both groups (Figure 1C). The relative abundances of *Lactobacillu*s and *Bifidobacterium* were remarkedly increased (*P* < 0.05) in the 1.5% XOS group (Figure 1D), while the relative abundances of *g_Clostridia_UCG-014, g__Ruminococcus, g__norank_f__Ruminococcaceae*, and *g__norank_f__Eubacterium_coprostanoligenes_group* in the 1.5% XOS group were markedly lower (*P* < 0.05) than the control group (Figure 1D). The differential bacteria were identified by using linear discriminant analysis (LDA) effect size (LEfSe). A dietary supplemented with 1.5% XOS was associated with increased relative abundances of *Lactobacillu*s, *Bifidobacterium*, and *Fusicatenibacter* at the genus level in weaning piglets (Figure 1E, LDA score >2).

### Quantification of Specific Bacteria in Feces

**Figure 2:**
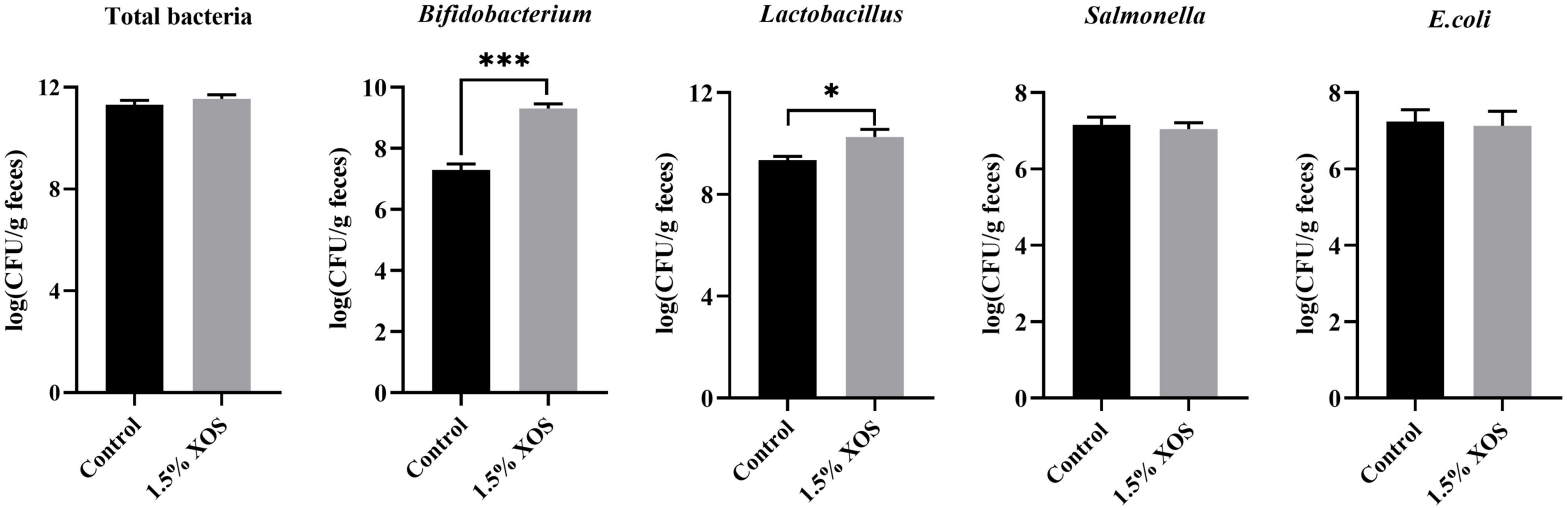
Effect of XOS on specific bacteria in feces. Data are presented as means + SEM. *P < 0.05 and ***P < 0.001, *n* = 6 for each group.

Compared with the control group, the pigs fed with a diet addition of 1.5% XOS markedly increased (*P* < 0.05) the copy numbers of *Bifidobacterium* and *Lactobacillus* in feces (Figure 2). However, 1.5% XOS did not differ (*P* > 0.05) the total bacteria, *Salmonella*, and *E. coli* (Figure 2).

### Effects of XOS Supplementation on Fecal SCFAs

The fecal SCFAs concentrations in response to different dosages of XOS supplementation on d 28 are shown in Table 8. Linearly increasing responses (*P* < 0.05) to the fecal acetate, propionate, butyrate, and total SCFAs were observed with the increasing supplemental XOS level. A dietary supplemented with 3% XOS had higher (*P* < 0.05) concentrations of acetate and propionate in feces than the control group, while there were no differences among in control, 0.75%, and 1.5% XOS groups or among the XOS treatments. Besides that, the supplementation of XOS markedly increased (*P* < 0.05) concentrations of butyrate and total SCFAs in feces compared with the control group, but no differences were noted in the XOS-treated groups (*P* > 0.05).

**Table 8:** The effects of dietary supplementation with XOS on SCFAs concentration in feces of weaning piglets.

**Items**	**XOS (% of diet)**					***P*** **-valve**		
	**Control (0)**	**0.75**	**1.5**	**3**	**SEM**	**Diet**	**Liner**	**Quadratic**
Acetate (mmol/kg)	130.80^b^	149.10^ab^	151.63^ab^	165.18^a^	4.23	0.026	0.004	0.458
Propionate (mmol/kg)	76.38^b^	91.09^ab^	90.64^ab^	99.92^a^	2.90	0.025	0.006	0.379
Butyrate (mmol/kg)	43.16^b^	59.67^a^	61.85^a^	68.97^a^	3.20	0.020	0.005	0.517
Total SCFAs (mmol/kg)	250.34^b^	299.86^a^	304.12^a^	334.08^a^	9.39	0.007	0.001	0.373

*SCFAs, short-chain fatty acids; SEM, standard error of the means*.

*Total SCFAs include acetate, prorate, and butyrate (n = 6 for each group). Values with different letter superscripts mean significant difference (P < 0.05) in a row, while with no letter superscripts mean no significant difference (P > 0.05)*.

### Correlation of Fecal Microbiota With Serum Parameters, SCFAs, and Growth Performance

**Figure 3:**
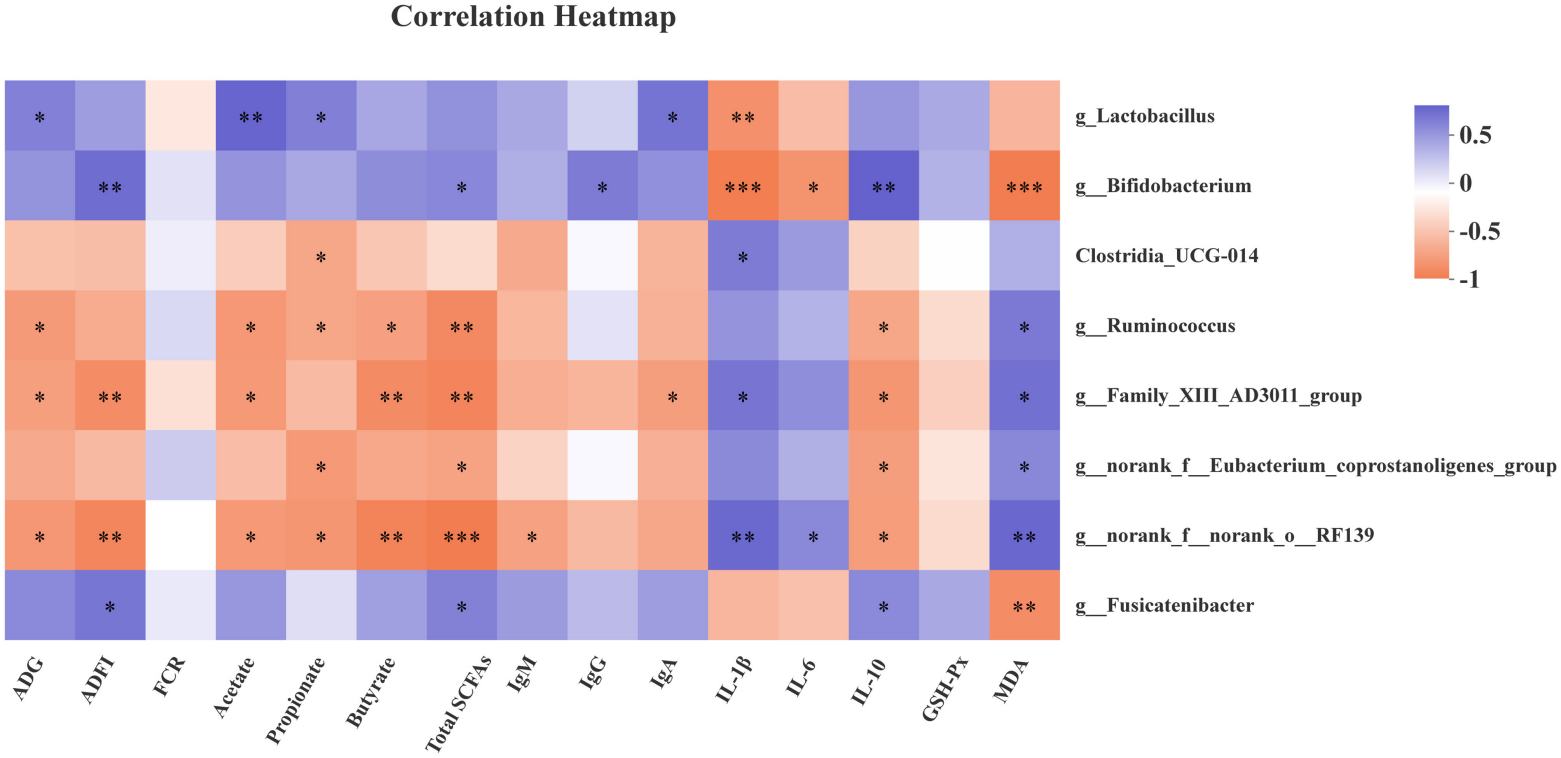
Correlation heatmap between microbiota and antioxidant capability, immune function, growth performance, and fecal SCFAs. Spearman correlations were applied. ADG, average daily gain; ADFI, average daily feed intake; FCR, feed conversion rate; Total SCFAs, total short-chain fatty acids; GSH-Px, glutathione peroxidase; MDA, malondialdehyde. Total SCFAs = acetate + propionate + butyrate. *P < 0.05, **P < 0.01, and ***P < 0.001.

As shown in Figure 3, the genus *Lactobacillu*s abundance was positively correlated with ADG, acetate, propionate, and IgA (*P* < 0.05), but negatively correlated with IL-1β (*P* < 0.05). The genus *Bifidobacterium* was positively related with ADFI, total SCFAs, IgG, and IL-10 (*P* < 0.05), as well as *g*_*Fusicatenibacter* abundance with ADFI, total SCFAs, and IL-10 (*P* < 0.05). However, the genus *Bifidobacterium* and *Fusicatenibacter* abundances were negatively associated with MDA (*P* < 0.05). Meanwhile, the genus *Bifidobacterium* abundance was negatively associated with IL-1β and IL-6 (*P* < 0.05). The genus *g_Ruminococcus, g__Family_XIII_AD3011_group*, and *g__norank_f__norank_o__RF39* were negatively correlated with ADG, acetate, propionate, and butyrate. In addition, there was a positive correlation between *g_Ruminococcus* and MDA level (*P* < 0.05), while a remarkable negative correlation was found between *g_Ruminococcus* and total SCFAs, and IL-10 levels (*P* < 0.05). There were similar results for *g__Family_XIII_AD3011_group, g__norank_f__norank_o__RF39, and g__norank_f__Eubacterium_coprostanoligenes_group*. Moreover, a positive correlation was observed between *g__norank_f__norank_o__RF39* abundance and IL-6 (*P* < 0.05), as well as *Clostridia*_*UCG-014, g__Family_XIII*_*AD3011_group, g__norank_f__norank_o__RF39* abundance, and IL-1β level.

## Discussions

Recently, the XOS as functional food are widely used in animal diets for improving animal growth performance, enhancing the quality of animal products, and regulating intestinal health (24). However, the abundance of *Bifidobacterium* did not show a remarkable increase at the low dosage of XOS in weaning pigs or growing pigs (4, 11, 18). In the present study, we investigated the effects of the high level of XOS on growth performance, antioxidant capability, and anti-inflammatory action, and whether the high level of XOS promotes *Bifidobacterium* abundance of weaning piglets. We found that the diet supplemented with 1.5% XOS significantly increased final BW, ADG, and ADFI during d 15–28 and the whole study period. Moreover, 28 d BW exhibited linear and quadratic responses (*P* < 0.05) with the increasing dietary XOS level, as well as ADFI on d 15–28, ADG on d 15–28 and 1–28, with the highest at the level of 1.5% XOS. As the dietary XOS level increased, ADFI on d 1–14 and 1–28 increased quadratically. Besides, the FCR on d 1–14 and 1–28 decreased linearly with increasing level of XOS in the diets. The 3% XOS group piglets had a lower ADFI and ADG than the 1.5% XOS group, which due to an excessive level of XOS may affect the dietary palatability. The previous studies showed that supplementation of XOS improved animal growth performance. Chen et al. (4, 13) found that an addition of 500 mg/kg XOS significantly increased BW, ADG, and decreased FCR in weaning piglets. Similarly, a dietary supplementation with 400 mg/kg XOS also increased final BW and ADG in nursery pigs, while ADFI and FCR were not affected (12). Additionally, a dietary supplementation of 250 mg/kg XOS markedly increased ADG and ADFI for weaning piglets (25). The difference between our findings and previous reports might be attributable to the dosage of XOS.

It has been reported that oligosaccharides may influence serum biochemical parameters (26). Our results showed that either the high level or the low level of XOS failed to affect serum biochemical parameters in weaning pigs. Consistently, the previous studies indicated that serum biochemical parameters were not altered in XOS-treated weaning pigs or limited effect in XOS-treated nursery pigs (11, 12, 27).

The CAT, SOD, and GSH-Px as the main antioxidant enzymes in the antioxidant protection system play a vital role in scavenging free radicals, reducing and eliminating oxidative damage. The serum T-AOC is an important indicator for evaluating the overall function of antioxidant capability *in vivo*. The MDA is considered as a biomarker for lipid peroxidation under the oxidative stress status. In this study, serum GSH-Px concentration increased linearly with increasing supplemental XOS level in weaning pigs. In addition, there were linear and quadratic responses between serum MAD level and XOS, with the lowest at the 1.5% XOS. The XOS had no impact on serum T-SOD, CAT, and T-AOC in our findings. Consistently, dietary addition of 400 mg/kg XOS markedly declined the serum MDA level and increased a trend of GSH-Px (12). Chen et al. (13) also reported that piglets fed with the diet of 500 mg/kg XOS had higher T-SOD and CAT levels on day 28, whereas the MDA level was lower than the control group. Moreover, T-SOD, T-AOC, and CAT levels exhibited quadratic increases with different dietary XOS levels on day 28, but the level of MDA was decreased quadratically. A recent report also found that maternal supplementation of 500 mg/kg XOS in diet had a lower level of MDA in sucking pigs, while did not affect the SOD, T-AOC, and CAT activity (28). These results indicated that XOS improvement of growth performance of weaned piglets might be related to increasing the antioxidant capability.

The immunoglobulins, including IgG, IgM, and IgA involve host immune response and protect against pathogens and virus infection. The previous studies showed that XOS can elevate the serum level of IgG in nursery pigs and IgM in broilers (12, 29). Notably, with the level of XOS increased, the serum IgA had a linear increase and IgM showed linear and quadratic responses in laying hens (30). Besides, 500 mg/kg XOS increased the concentration of serum IgG on day 28 in weaning piglets, and serum IgG concentration showed a quadratic effect with increasing XOS level in diet (13). Similarly, our results found that a dietary supplementation of 0.75 and 1.5% XOS significantly increased the serum IgM concentration in weaning piglets compared with the control group. Additionally, 1.5% and 3% XOS had higher levels of IgG than the control group. Moreover, the serum level of IgA was increased later in XOS-treated pigs. Numerous studies indicated that XOS can alleviate inflammatory status in animals. Sow supplementation of 500 mg/kg XOS decreased serum IL-1β, IL-6, IL-2, and Tumor Necrosis Factor (TNF)-α levels in sucking piglets (28). In the present study, 1.5 and 3% XOS significantly decreased the serum IL-1β compared with the control group. The XOS-treated piglets had a lower serum level of IL-6 than the vehicle group. Furthermore, anti-inflammatory cytokine IL-10 was markedly increased in the 1.5% XOS group. Notably, the effects of XOS supplementation could depend on the dosage, since Yin et al. (11) found XOS at 100 mg/kg only decreased the serum IFN-γ in weaning piglets, but had no impacts on IL-1β, IL-6, and IL-10. Hansen et al. (31) reported that a diet supplemented with 10% XOS downregulated the IFN-γ and IL-1β in mice. Importantly, XOS suppressed the pro-inflammatory cytokines, IL-1β, IL-6, and TNF-α secretion from RAW265.7 macrophages stimulated with lipopolysaccharide in the pretreatment model (32). The possible mechanism responsible for the beneficial effects is that XOS can enhance immune function and decrease the inflammatory status in weaning piglets.

It has been widely demonstrated that XOS can modulate gut microbiota composition by selectively stimulating beneficial bacteria. Thus, the microbiota composition of fecal samples was analyzed by 16S rRNA gene sequencing. The result found that 1.5% XOS significantly decreased the Shannon index and increased the Simpson index, indicating dietary supplementation of XOS decreased the diversity of gut microbiota community. Similarly, the addition of 250 mg/kg XOS significantly decreased the Shannon index during growing fatty pigs (16). The XOS decreased α-diversity Chao1 and Shannon indices in high-fat diet-induced mice as well (33). The α-diversity decrease might result from increasing the relative abundances of *Lactobacillus* and *Bifidobacterium* in XOS-treated pigs. The PCoA plots based on Bray-Curtis distances were a clear separation between XOS and control groups, which is similar to that reported by Pan et al. (16). It has been reported that XOS can promote counts of *Lactobacillus* in weaning piglets (4, 16), while the low level of XOS failed to stimulate the abundance of *Bifidobacterium* (4, 11, 18, 34). In the current study, the abundances of *Lactobacillus* and *Bifidobacterium* were markedly increased in 1.5% XOS-treated pigs using l6S rRNA gene sequencing, as well as by qPCR. Notably, 1.5% XOS elevated the relative *Bifidobacterium* abundance approximately 35-fold from 0.021 to 0.74%. The high level of XOS did not markedly vary *Bifidobacterium* abundance in weaning piglets compared to rodents (17), probably due to the differences of microbial stability and physiological backgrounds between animal species. Holman et al. (35) analysis of core microbiota in the swine gut revealed that *Clostridium, Blautia, Lactobacillus, Prevotella, Ruminococcus, Roseburia*, the RC9 gut group, and *Subdoligranulum* were considered as core microbiota but the relative abundance of *Bifidobacterium* in fecal samples remained low (<0.35%). Moreover, the XOS treatments linearly increased the fecal acetate, propionate, butyrate, and total SCFAs levels in weaning piglets. Chen et al. (4) also found that the dietary supplementation of 500 mg/kg XOS elevated the acetate, propionate, butyrate, and total SCFAs concentrations in the cecum of piglets. Furthermore, the *Lactobacillu*s, *Bifidobacterium*, and *Fusicatenibacter* were regarded as biomarkers in XOS-treated pigs identified by LEfSe analysis (LDA > 2). Collectively, the high level of XOS can alter the fecal microbiota composition and increase the concentrations of SCFAs by promoting the beneficial bacteria.

The growing evidence has shown that *Lactobacillus* and *Bifidobacterium* are associated with SCFAs production, antioxidant activity, and host inflammatory status (36–39). Thus, spearman correlations between bacteria and antioxidant, immune function, growth performance, and fecal SCFAs were performed. The *Lactobacillus* exhibited positive correlations with ADG, acetate, propionate, and serum IgA level, but a negative correlation with serum IL-1β level. The *Bifidobacterium* was positively associated with ADFI, total SCFAs, IgG, and IL-10, while negatively associated with levels of IL-1β, IL-6, and MDA in serum. The recent reports indicated that *Fusicatenibacter* is a SCFAs-producing bacteria, which closely related to host health, such as Parkinson's disease, ulcerative colitis (40–44). The *Fusicatenibacter* suppresses intestinal inflammation and is positively associated with SCFAs production (41). Consistently, there were positive correlations between *Fusicatenibacter* and ADFI, total SCFAs, and IL-10 level, while a negative correlation was observed between *Fusicatenibacter* and MDA in our findings. However, the bacteria with higher abundances in the control group, like *g_Ruminococcus, g__Family_XIII_AD3011_group*, and *g__norank_f__norank_o__RF39*, were in negative relations with ADG, ADFI, SCFAs, and IL-10, and in positive relations with IL-1β and MDA.

## Conclusions

In conclusion, our results indicated that XOS supplementation improved the growth performance, increased antioxidant capability, enhanced immune function, and decreased body inflammatory status and incidence of diarrhea in weaning piglets, which could be contributed to the modulation of gut microbiota community, especially by increasing the abundances of *Lactobacillus, Bifidobacterium*, and *Fusicatenibacter*. However, the high level of XOS did not sharply the relative abundance of *Bifidobacterium* in feces. The optimum level of XOS supplementation was 1.5% for weaning piglets in this study.

## Data Availability Statement

The datasets presented in this study can be found in online repositories. The names of the repository/repositories and accession number(s) can be found below: NCBI SRA; PRJNA762151.

## Ethics Statement

The animal study was reviewed and approved by Animal Ethics Committee of China Agricultural University.

## Author Contributions

JP wrote the manuscript. JP, XZ, YW, and DL performed the experiments. HY revised the manuscript. ZW analyzed the data. JW obtained financial support and oversaw this study. JP, DH, and JW designed the research. All authors contributed to the article and approved the submitted version.

## Funding

This research was funded by the National Natural Science Foundation of China (31972596, 31630074, 31902170), the Beijing Municipal Natural Science Foundation (S170001), the China Agriculture Research System (CARS-35), and the Fundamental Research Funds for the Central Universities (2021TC089).

## Conflict of Interest

The authors declare that the research was conducted in the absence of any commercial or financial relationships that could be construed as a potential conflict of interest.

## Publisher's Note

All claims expressed in this article are solely those of the authors and do not necessarily represent those of their affiliated organizations, or those of the publisher, the editors and the reviewers. Any product that may be evaluated in this article, or claim that may be made by its manufacturer, is not guaranteed or endorsed by the publisher.
